# CBX3 Regulated By YBX1 Promotes Smoking-induced Pancreatic Cancer Progression via Inhibiting SMURF2 Expression

**DOI:** 10.7150/ijbs.68995

**Published:** 2022-05-13

**Authors:** Huan Zhang, Haixin Yu, Dianyuan Ren, Yan Sun, Feng Guo, Hongkun Cai, Chen Zhou, Yingke Zhou, Xin Jin, Heshui Wu

**Affiliations:** 1 Department of Pancreatic Surgery, Union Hospital, Tongji Medical College, Huazhong University of Science and Technology, Wuhan, 430022, China; 2 Sino-German Laboratory of Personalized Medicine for Pancreatic Cancer, Union Hospital, Tongji Medical College, Huazhong University of Science and Technology, Wuhan 430022, China; 3 Cancer center, Union Hospital, Tongji Medical College, Huazhong University of Science and Technology, Wuhan, 430022, China; 4 Department of Urology, The Second Xiangya Hospital, Central South University, Changsha, Hunan, 410011, China; 5 Uro-Oncology Institute of Central South University, Changsha, Hunan, 410011, China

**Keywords:** CBX3, Smoking, Pancreatic cancer, YBX1, SMURF2

## Abstract

As a key reversible and heritable mechanism of transcriptional regulation, the epigenetic modification plays a crucial role in tumorigenesis. Of note, tobacco smoking induces epigenetic modifications to promote pancreatic cancer development. Chromobox protein homolog 3 (CBX3) acts as an epigenetic regulator, modulating gene expression of downstream targets via chromatin modifications. To date, the relationship between CBX3 and smoking in pancreatic cancer remains unknown. This study aimed to uncover the specific role and underlying mechanism of CBX3 in smoking-related pancreatic cancer. The bioinformatics analyses were conducted to identify CBX3 as a key player in tobacco-induced pancreatic cancer. The abnormal upregulation of CBX3 was associated with poor prognosis in pancreatic cancer patients. Moreover, cigarette smoke extract (CSE) exposure promoted the overexpression of Y-box-binding protein 1 (YBX1), which consequently led to upregulated CBX3 in pancreatic cancer cells. We also revealed that CBX3 enhanced pancreatic cancer progression, likely by inhibiting the expression of SMAD specific E3 ubiquitin protein ligase 2 (SMURF2) and promoting the activation of TGF‐β signaling. In summary, the YBX1/CBX3/SMURF2 signaling axis may be a promising therapeutic target in patients with smoking-related pancreatic cancer.

## Introduction

Pancreatic cancer is a kind of lethal solid tumor and causes significant morbidity worldwide 1. Smoking is a known risk factor for pancreatic cancer development and progression, contributing to approximately 20% of all pancreatic cancer cases 2, 3. Recent evidence also suggests that smoking is associated with poor prognosis in patients with pancreatic cancer 4-6. However, the mechanisms underlying the correlation between smoking and pancreatic cancer development and progression remain elusive.

Epigenetic modifications consist of histone modifications (e.g. methylation and acetylation), DNA methylation and demethylation, and gene expression regulation by non-coding RNAs 7. Epigenetic modifications are a key reversible and heritable mechanism of transcriptional regulation 8 and play a crucial role in tumorigenesis 9. For instance, temporal or persistent smoking-induced DNA methylation is a hallmark of various cancers, such as lung cancer 10, 11. Furthermore, tobacco smoking-related genotoxicity can enhance disease progression in pancreatic cancer by modulating pivotal biological aspects, such as cell defense and DNA repair, as well as tobacco-carcinogen metabolization 12.

Polycomb repressive complexes surpress downstream target genes involved in various biological processes through epigenetic regulation 13. Chromobox (CBX) family proteins are key parts of Polycomb repressive complex 1 (PRC1) 14-16. The CBX family consists of eight members (CBX1-8) and is vitally important to tumorigenesis and malignant progression 16. The bioinformatic analysis indicated that Chromobox protein homolog 3 (CBX3) had a close relationship with tobacco-induced pancreatic cancer. However, the specific regulatory function and mechanism of CBX3 in smoking-related pancreatic cancer remain unknown.

Herein, this study was conducted to investigate the pivotal roles and potential mechanisms of CBX3 in smoking-related pancreatic cancer. We found that Y-box binding protein 1 (YBX1), as a smoking-related protein 17 and oncogenic driver 18, could enhance the transcription of CBX3 in pancreatic cancer cells. Furtherly, CBX3 could promote cell proliferation through the suppression of SMAD specific E3 ubiquitin protein ligase 2 (SMURF2) in pancreatic cancer. Altogether, these findings strongly suggest CBX3 as a promising therapeutic target in pancreatic cancer.

## Materials and Methods

### Data mining and bioinformatic analysis

The Clinical Proteomic Tumor Analysis Consortium (CPTAC), the International Cancer Genome Consortium (ICGC), the Cancer Genome Atlas (TCGA), Tumor immune estimation resource 2.0 (TIMER2.0), and ChIP-Atlas were used for data mining and bioinformatic analysis (see Supplementary Methods for details).

### Cell culture and Cell transfection

The pancreatic cancer cell lines SW1990, AsPC-1, MIAPaCa-2, CFPAC1, PANC-1, and BxPC-3 were purchased from ATCC (USA). All cell lines had the STR authentication. Cells were maintained in Dulbecco's Modified Eagle Media (DMEM, Thermo Fisher Scientific, USA) medium added with 10% fetal bovine serum (FBS, HyClone, USA) at 37 °C in a 5% CO2 incubator. Cell transfection with plasmids was carried out using Lipofectamine 2000 Reagent (Thermo Fisher Scientific, USA) following the manufacture s instructions. Small hairpin RNAs (shRNAs) targeting CBX3 and YBX1 and overexpression plasmid (GV657 vector, YBX1 and CBX3) were obtained from Shanghai Gene Chem. To construct the stably transfected cell lines, cells were transfected with gene-specific shRNAs or overexpression plasmids by using the Lipofectamine 2000 Reagent. And then, puromycin with corresponding concentration was used to select cell lines that had been successfully transformed by vectors that expressed puromycin-N-acetyl-transferase after transfection for 72h. After that the fluorescence distribution of green fluorescent protein (GFP) was next to 100%, we reduced the concentration of puromycin to half to keep the cell screening.

### Preparation of cigarette smoke extract

The cigarettes (Hongta, China, 1.0 mg nicotine per cigarette; 10 mg tar per cigarette) were selected. The smoke from burning 20 cigarettes was pumped into 200 ml DMEM serum-free medium. The pondus hydrogenii (pH) of the cigarette smoke extract (CSE) medium was adjusted to 7.4 and was filtrated with a sterile 0.22-μm filter. The 10% stock CSE medium (1 cigarette per 10 mL medium) was stored at -20℃ to keep concentration stable and then diluted to the working concentrations with DMEM medium.

### Western blot analysis

Briefly, cells were harvested and lysed by modified radio immunoprecipitation assay (RIPA) lysis buffer. The protein quantification kit (Sigma-Aldrich, USA) was used to determine the protein concentration. Proteins were loaded into the SDS-PAGE and transferred to PVDF membranes. Then, proteins were incubated with specific primary antibodies and detected by Enhanced Chemiluminescence (ECL) assay. Image J (National Institutes of Health, NIH) was applied to quantified the interested proteins.

### RNA Isolation and Quantitative RT-PCR

Total RNA was extracted using Trizol reagent (Invitrogen, USA), and reverse transcribed into complementary DNA using PrimeScript RT reagent kit (TakaRa, Japan). Then, we used TB Green™ Fast qPCR Mix kit (TakaRa, Japan) to carry out RT- PCR analysis. With the cyclic index normalized to GAPDH, we used the 2^-ΔΔCT^ method to calculate and analyse multiple variations.

### Chromatin immunoprecipitation (ChIP)-qPCR

A chromatin extraction kit (Abcam, USA) and the ChIP Kit Magnetic - One Step (Abcam, USA) were used to perform ChIP following the manufacturer's instructions reported previously. The primer sequences for ChIP-qPCR are provided in Tables S3.

### Dual-Luciferase Reporter Assay

The promoter (1000bp before the TSS, containing the whole binding peak of YBX1) of the CBX3 gene (GenBank: NM_016587) was amplified and cloned into the PGL3-basic (+) expression vector. The constructed plasmid (PGL3-CBX3 Pro) was verified by DNA sequencing. The indicated plasmid was transfected into cells by using the Lipofectamine 2000 Reagent. Forty-eight hours after transfection, cells were harvested and tested with the Dual-Luciferase Reporter Gene Assay Kit (Beyotime, China) following the manufacturer's instructions.

### Colony formation assay

Biological effect of CBX3 and SMURF2 on cancer cell survival was evaluated by colony formation assay. We seeded pancreatic cancer cells into the 6-well plates with 500 cells per well. After 14 days, cell colonies were fixed by 4% paraformaldehyde for 30 minutes followed by crystal violet for 20 minutes.

### MTS assay

Cell viability was measured by absorbance using the MTS (3-(4,5dimethylthiazol-2-yl)-5-(3-carboxymethoxyphenyl)-2-(4sulfophenyl)-2H-tetrazolium, inner salt) assay according to the manufacturer's instructions (Abcam, USA). Briefly, the pancreatic cancer cells were seeded at a density of 1,000 cells per well in 96-well plates with 100μl of DMEM medium. The cells were treated with serial concentrations of small molecular inhibitors. At the indicated point-in-time, 20µl of MTS reagent (Abcam, USA) was added to each well of the cells and incubated for 1 h at 37°C in an incubator positioned away from the light. The absorbance was assessed in a microplate reader at 490 nm.

### Tissue microarray and immunohistochemistry (IHC)

Tissue microarray slides were purchased from Outo Biobank (Shanghai, China). These slides were immunostained with CBX3 (Proteintech, working dilution 1:2000), SMURF2 (Affinity Biosciences, working dilution 1:1500). The staining index (SI) was calculated as the product of the staining intensity score and the proportion of positive tumor cells. The staining intensity was graded according to the following criteria: “1” means “weak staining at 100×magnification but little or no staining at 40× magnification”; “2” means “medium staining at 40×magnification”; “3” means “strong staining at 40× magnification”. The immunostaining was scored independently by two experienced pathologists.

### PDAC xenografts in nude mice

The BALB/c-nude mice (4-5 weeks old, 15-20g) were purchased from Vitalriver (Beijing, China). The stably transfected cell lines (5×10^6^ pancreatic cancer cells per mouse) were subcutaneously inoculated on the left back side of mice. The length and width of xenografts was measured by vernier caliper and their volumes were calculated by the formula (L×W^2^)/2. After about 3 weeks, all mice were euthanized and then all xenografts were excised to weight. All animal experiment procedures were approved by the Ethics Committee of Tongji Medical College, Huazhong University of Science and Technology.

### Transwell assay

Pancreatic cancer cells were cultured into the 24-well Transwell plates with 8.0 µm pores (Corning Costar, USA) with precoated Matrigel (BD, USA; diluted 1: 8). Briefly, 5×10^4^ pancreatic cancer cells were seeded into the top chamber. After cultivation for 24 hours, the membrane were collected and stained with the Crystal Violet Solution (Solarbio, China). A cotton swab was used to remove those cells that did not migrate or invade through the pores. The migrating cells were counted and photographed by a microscope from five different microscopic fields per well.

### Statistical analysis

All data were presented as the means ± standard deviation (SD). Statistical significance was determined by student T test, one-way and two-way ANOVA using GraphPad Prism 8 software. P-values <0.05 were considered statistically significant.

Other unmentioned methods were provided in the Supplementary information.

## Results

### Smoking-induced CBX3 upregulation is correlated with poor prognosis in pancreatic cancer

**Fig 1:**
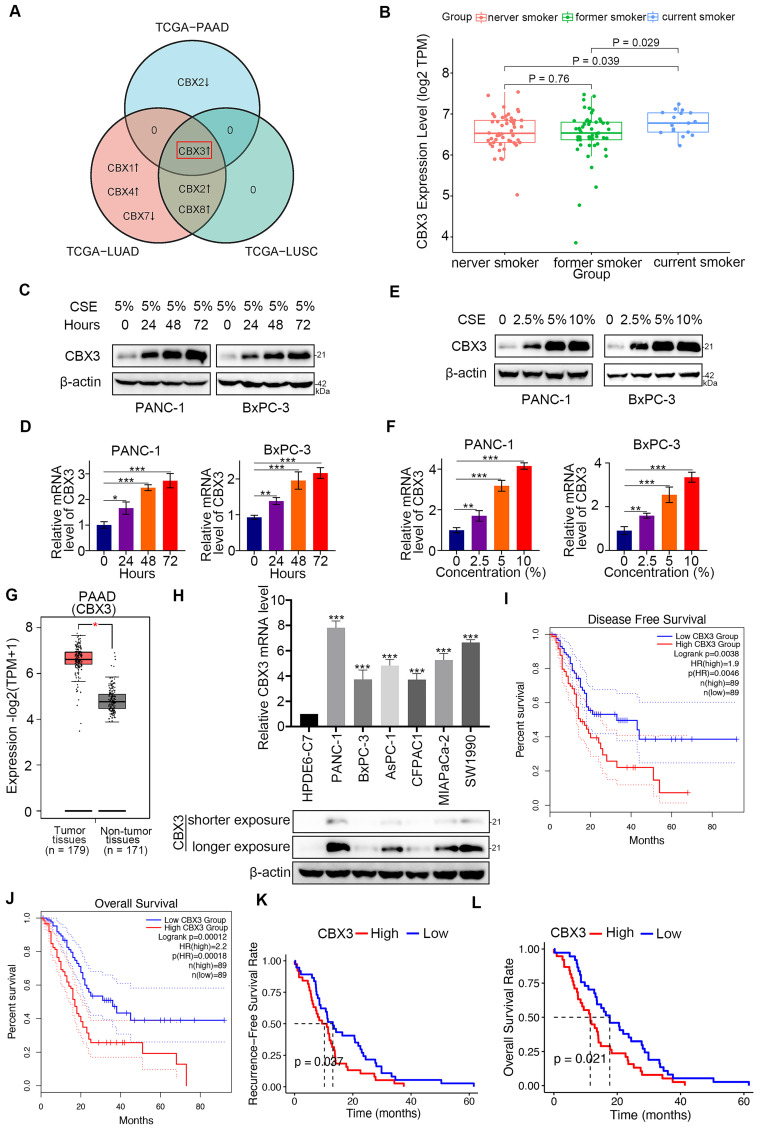
** Smoking-induced CBX3 upregulation is correlated with poor prognosis in pancreatic cancer. (A)** Venn diagrams showed numbers of differentially expressed CBX family genes in current smokers compared to former smokers in TCGA-PAAD (Pancreatic adenocarcinoma), TCGA-LUAD (Lung adenocarcinoma) and TCGA-LUSC (Lung squamous cell carcinoma) datasets. **(B)** Box plot showed CBX3 was upregulated in current smokers compared to former smokers or never smokers in TCGA-PAAD dataset. **(C and D)** PANC-1 and BxPC-3 cells were treated with CSE (5%) for 0, 24, 48 and 72 hours as indicated, and then were collected for Western blot (C) and RT-qPCR assay (D). **(E and F)** PANC-1 and BxPC-3 cells were treated with a serial concentration of CSE for 72 hours, and then were collected for Western blot (E) and RT-qPCR assay (F). **(G)** The tissue-wise expression of CBX3 in Pancreatic adenocarcinoma (PAAD) tissues and non-tumor tissues were analyzed by the GEPIA web tool. P < 0.01*. **(H)** The mRNA and protein levels of CBX3 were examined. **(I and J)** CBX3-related Disease Free Survival (I) and Overall Survival (J) were determined by the GEPIA web tool. **(K and L)** CBX3-related Recurrence Free Survival (K) and Overall Survival (L) were checked in the ICGC-PACA-AU dataset. Data in (D), (F) and (H) are presented as mean ± SD (n = 3). P < 0.05 *; P < 0.01 **; P < 0.001 ***.

Smoking is a key environmental risk factor leading to epigenomic alterations in pancreatic cancer 19. CBX family members epigenetically repress the gene expression of PRC1 complex 20. Here, we explored the role of CBX family proteins in smoking-related pancreatic cancer. Interestingly, bioinformatic analysis revealed that among the eight CBX family members, only CBX3 was upregulated in both smoking-related pancreatic cancer and lung cancer (Fig. 1A). We divided patients with pancreatic cancer into three groups: current smokers, former smokers, and never smokers, and found that CBX3 was markedly increased in current smokers (*P* < 0.05; Fig. 1B and Fig. S1A-G). Additionally, in vitro analyses indicated that the expression of CBX3 was increased by CSE exposure in a time-dependent and dose-dependent manner (Fig. 1C-F).

We also analyzed the clinical relevance of CBX3 and other CBX family members in pancreatic cancer. Bioinformatic analyses through the GEPIA web tool revealed that CBX3, CBX1, and CBX5 were significantly overexpressed in the pancreatic cancer tissues than those in non-malignant pancreatic tissues (*P* < 0.01) (Fig. 1G and Fig. S1H-N). Moreover, the expression of CBX3 were higher in pancreatic cancer cell lines than normal human pancreatic duct epithelial cells (HPDE6-C7) (Fig. 1H). Notably, the high expression of CBX3 was correlated with poor prognosis (disease free survival and overall survival) in pancreatic cancer patients from the TCGA dataset (Fig. 1I-J; Fig. S2). Consistently, survival analyses of the ICGC dataset further supported CBX3 as a poor prognostic predictor in pancreatic cancer (Fig. 1K-L). These data suggests that cigarette smoking increases the expression of CBX3, and consequently contributes to poor clinical outcomes in pancreatic cancer.

### YBX1 enhances CBX3 transcription in smoking-related pancreatic cancer

**Fig 2:**
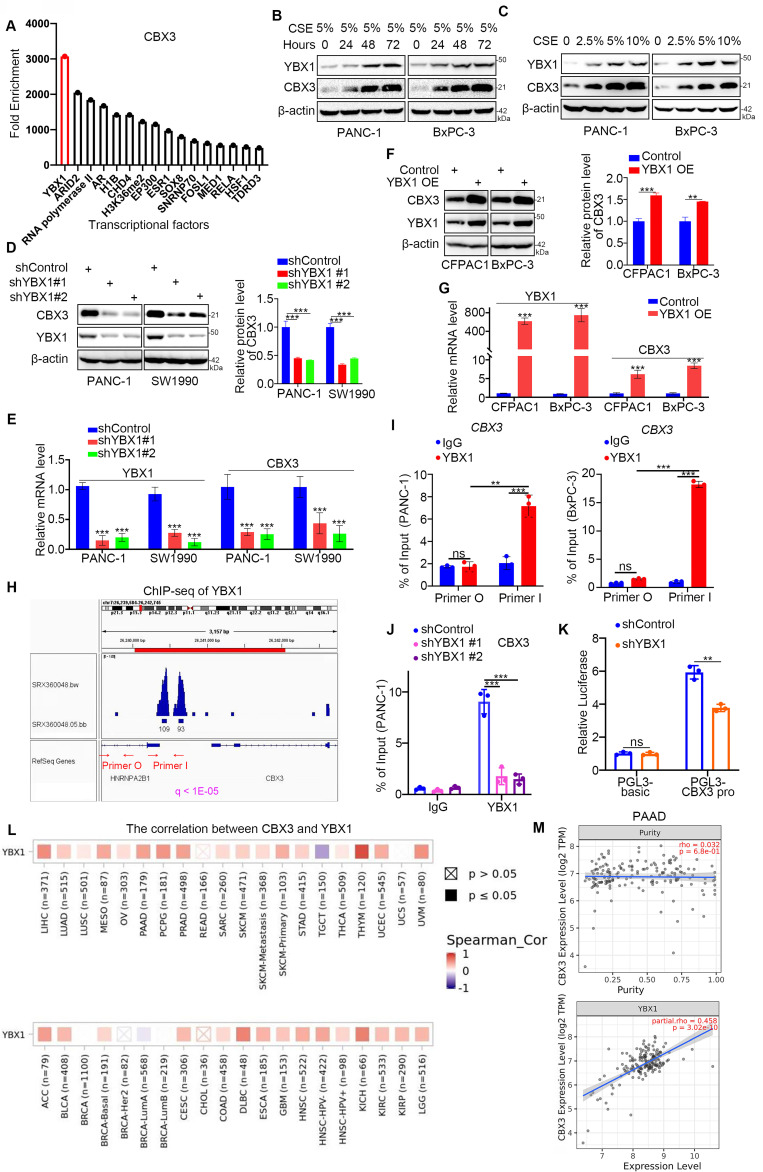
** YBX1 increases the expression of CBX3 through transcriptional regulation in smoking related pancreatic cancer. (A)** Enrichment analysis by ChIP-Atlas predicted a large amount of YBX1 bound to promoter region of CBX3 within ±1000bp from TSS. **(B)** PANC-1 and BxPC-3 cells were treated with CSE (5%) for 0, 24, 48 and 72 hours as indicated, and then were collected for Western blot. **(C)** PANC-1 and BxPC-3 cells were treated with a serial concentration of CSE for 72 hours, and then were collected for Western blot. **(D and E)** PANC-1 and SW1990 cells were transfected with indicated shRNAs for 72 hours. After puromycin selection, cells were harvested for the Western blot (D) and RT-qPCR assay (E). The protein level of CBX3 was quantified by the Image J software. **(F and G)** CFPAC1 and BxPC-3 cells were transfected with indicated plasmids for 24 hours, and then were harvested for Western blot (F) and RT-qPCR assay (G). The protein level of CBX3 was quantified by the Image J software. **(H)** The ChIP-seq data of YBX1 indicated that there were binding peaks in the promoter region of CBX3. **(I)** The ChIP-qPCR assay of CBX3 by using the IgG or YBX1 antibodies in the PANC-1 and BxPC-3 cells. **(J)** PANC-1 cells were transfected with indicated shRNAs for 72 hours, and then were harvested for the ChIP-qPCR assay by using the IgG or YBX1 antibodies. **(K)** The double luciferase report assay was conducted to investigate whether YBX1 could regulate the promoter activities of CBX3 (Fig. 2K).** (L and M)** TIMER2.0 showed purity-adjusted correlation between CBX3 and YBX1 in various cancer types (K) and PAAD, exclusively (L). Data in (D), (E), (F), (H), (I) and (J) are presented as mean ± SD (n = 3). Not significant ^Ns^; P < 0.05 *; P < 0.01 **; P < 0.001 ***.

To explore the mechanisms driving CBX3 upregulation in response to smoking, we analyzed chromatin immunoprecipitation (ChIP)-seq data and identified several transcriptional factors (TFs) that could bind to the promoter region of CBX3. YBX1 was identified as the TF with the highest enrichment in the promoter region of CBX3. (Fig. 2A). It has been reported that cigarette smoking could increase the mRNA levels of YBX1 in mice 17. Consistently, YBX1 was obviously upregulated by CSE exposure in pancreatic cancer cells (Fig. 2B-C), suggesting a potential role of YBX1 in smoking-induced CBX3 expression in pancreatic cancer. To test this hypothesis, we knocked down YBX1 using shRNAs, and found that YBX1 silencing reduced CBX3 expression in PANC-1 and SW1990 cells (Fig. 2D-E). Conversely, the overexpression of YBX1 could upregulate CBX3 in CFPAC1 and BxPC-3 cells (Fig. 2F-G). ChIP-seq analysis showed a binding peak of YBX1 in the promoter region of CBX3 (Fig. 2H). ChIP-qPCR data confirmed that YBX1 could bind to the promoter region of CBX3 (Fig. 2I-J). The double luciferase report assay further indicated that YBX1 could enhance the promoter activities of CBX3 (Fig. 2K). Moreover, bioinformatic analyses indicated a positive correlation between YBX1 and CBX3 in the mRNA level in various cancers (Fig. 2L), including pancreatic cancer (Fig. 2M). These results suggest that YBX1 could promote the upregulation of CBX3 at the transcriptional level in pancreatic cancer cells.

### CBX3 enhances malignant potential of pancreatic cancer

**Fig 3:**
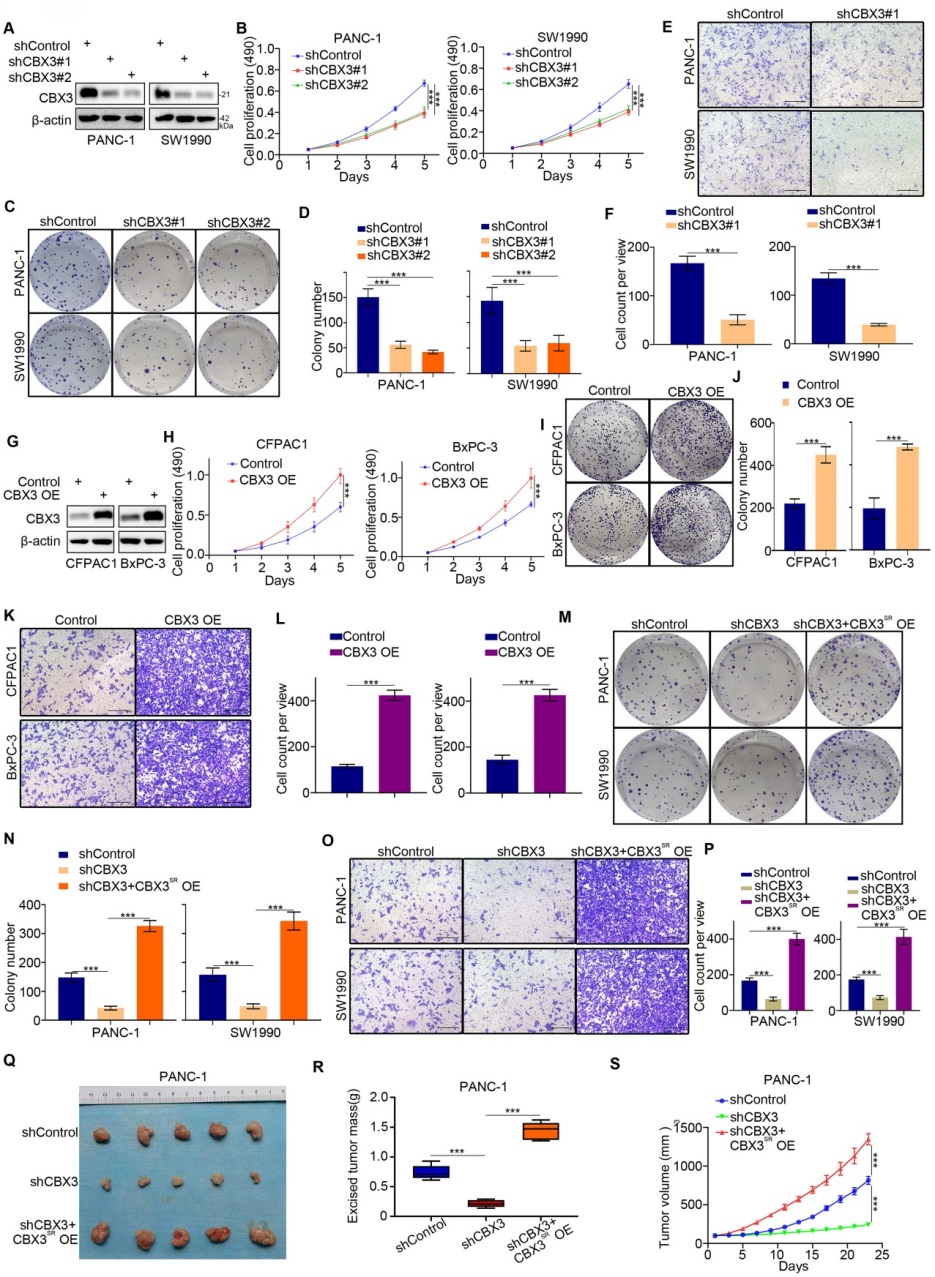
** CBX3 is critical for the malignant progression of pancreatic cancer. (A-F)** PANC-1 and SW1990 cells were transfected with CBX3 shRNAs for 72 hours. After puromycin selection, cells were harvested for the Western blot (A), MTS assay (B), colony formation (C and D), and transwell assay (E and F). **(G-L)** CFPAC1 and BxPC-3 cells were transfected with indicated constructs for 72 hours. After puromycin selection, cells were harvested for the Western blot (G), MTS assay (H), colony formation (I and J), and transwell assay (K and L). **(M-S)** PANC-1 and SW1990 cells were infected with indicated shRNAs for 48 hours. Then, these cells were infected with shRNA-resistant overexpression (CBX3^SR^ OE) construct for another 72 hours. After puromycin selection, cells were harvested for the colony formation (M and N), transwell assay (O and P), and subcutaneous xenograft assay (Q-S). Data in (B), (D), (F), (H), (J), (L), (N), (P), (R) and (S) are presented as mean ± SD (n = 3); P < 0.001 ***.

As CBX3 overexpression was a poor prognostic predictor, we next evaluated the biological characteristics of CBX3 in pancreatic cancer cells. CBX3 knockdown inhibited pancreatic cancer cell proliferation (Fig. 3A-D) and invasion (Fig. 3E-F). Conversely, CBX3 overexpression promoted proliferation and invasion of pancreatic cancer cells (Fig. 3G-L). Rescuing the expression of CBX3 in CBX3-knockdown cells restored the abilities of growth and invasion (Fig. 3M-P). Subcutaneous injection of pancreatic cancer cells in mice was performed to study the role of CBX3 in vivo. CBX3 silencing suppressed tumor growth, and rescuing CBX3 expression attenuated this inhibitory effect (Fig. 3Q-S). Thus, these results suggest that CBX3 plays a vital part in disease progression of pancreatic cancer. Moreover, further MTS assay and Transwell assay indicated that YBX1 promoted pancreatic cancer cell proliferation and invasion by upregulating CBX3 (Fig. S3A-D).

### CBX3 activates the TGF-β pathway and downregulates SMURF2 in pancreatic cancer

**Fig 4:**
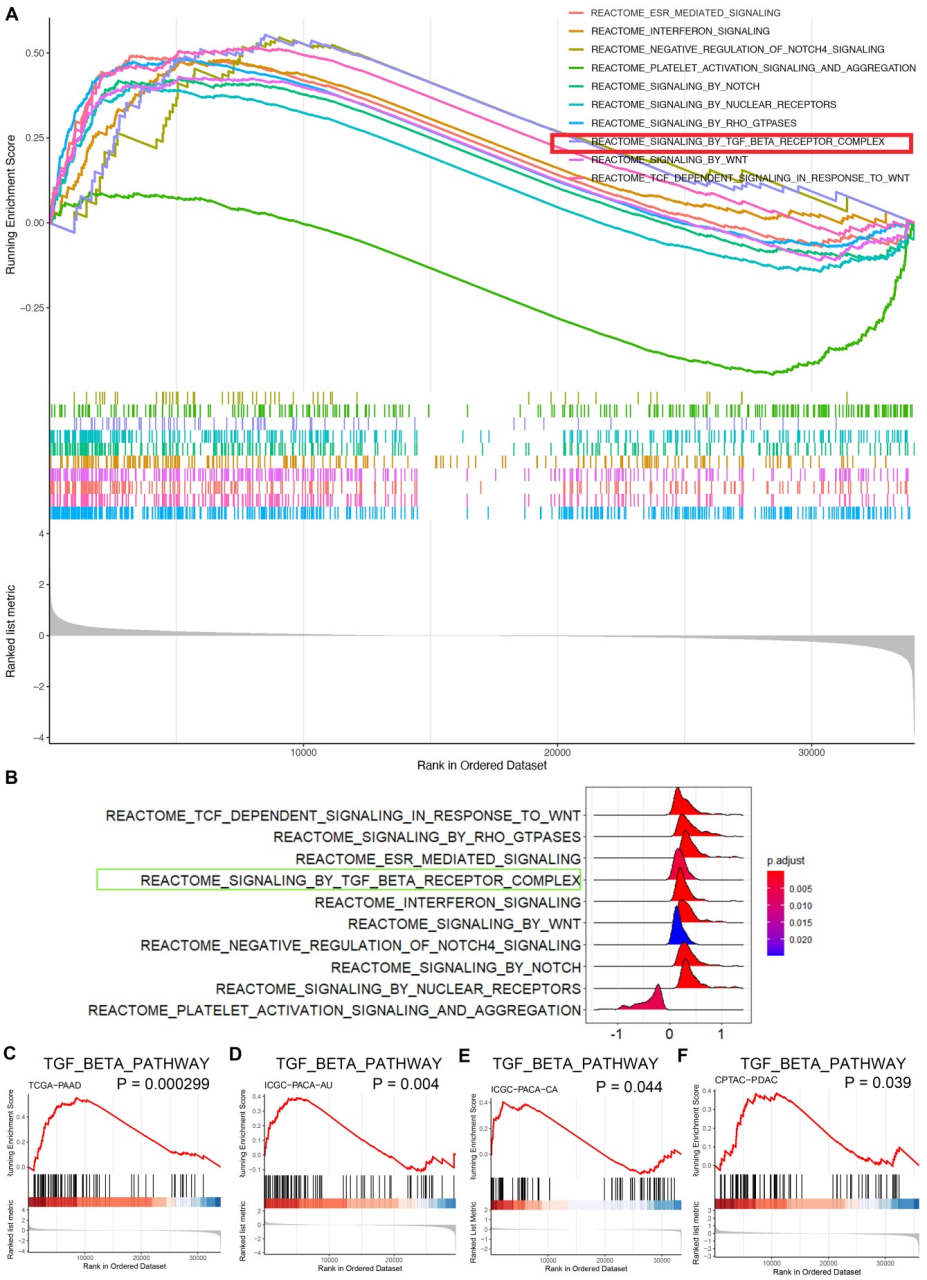
** CBX3 triggers the hyperactivation of TGF-**β** signaling pathway in pancreatic cancer. (A and B)** GSEA indicated top 10 REACTOME signaling pathways induced by the overexpression of CBX3 in TCGA-PAAD dataset. Multi-GSEA plot showed running enrichment score for gene sets as the analyses walk down the preranked gene list (A). Ridge plot showed density of signaling pathway generated by using the frequency of fold change values per gene within each set (B). **(C-F)** REACTOME signaling by TGF-beta receptor complex was activated by the overexpression of CBX3 in TCGA-PAAD (C), ICGC-PACA-AU (D), ICGC-PACA-CA (E) and CPTAC-PDAC (F) datasets (all P < 0.05*).

To further investigate the mechanisms underlying the role of CBX3 in pancreatic cancer aggressiveness, bioinformatic analyses based on TCGA dataset revealed that CBX3 was closely associated with several oncogenic signaling pathways (Fig. 4A-B). Next-generation sequencing of cancer tissues showed that pancreatic cancer was highly heterogeneous with multiple gene mutations. Apart from mutations in Kras and TP53, SMAD4 mutations were also common and found in half of pancreatic cancer patients. Such SMAD4 mutations could trigger the hyperactivation of the TGF-β signaling pathway 21, indicating the vital role of this pathway in pancreatic cancer progression. Notably, the TGF-β signaling was predicated to be one of the key pathways activated by CBX3 through gene set enrichment analysis (GSEA) (Fig. 4A-C). Additionally, GSEA based on the datasets ICGC-PACA (Pancreatic Cancer)-AU (Australia), ICGC-PACA-CA (Canada) and CPTAC-PDAC also demonstrated that CBX3 played an essential role in activating the TGF-β signaling pathway in pancreatic cancer (Fig. 4D-F).

**Fig 5:**
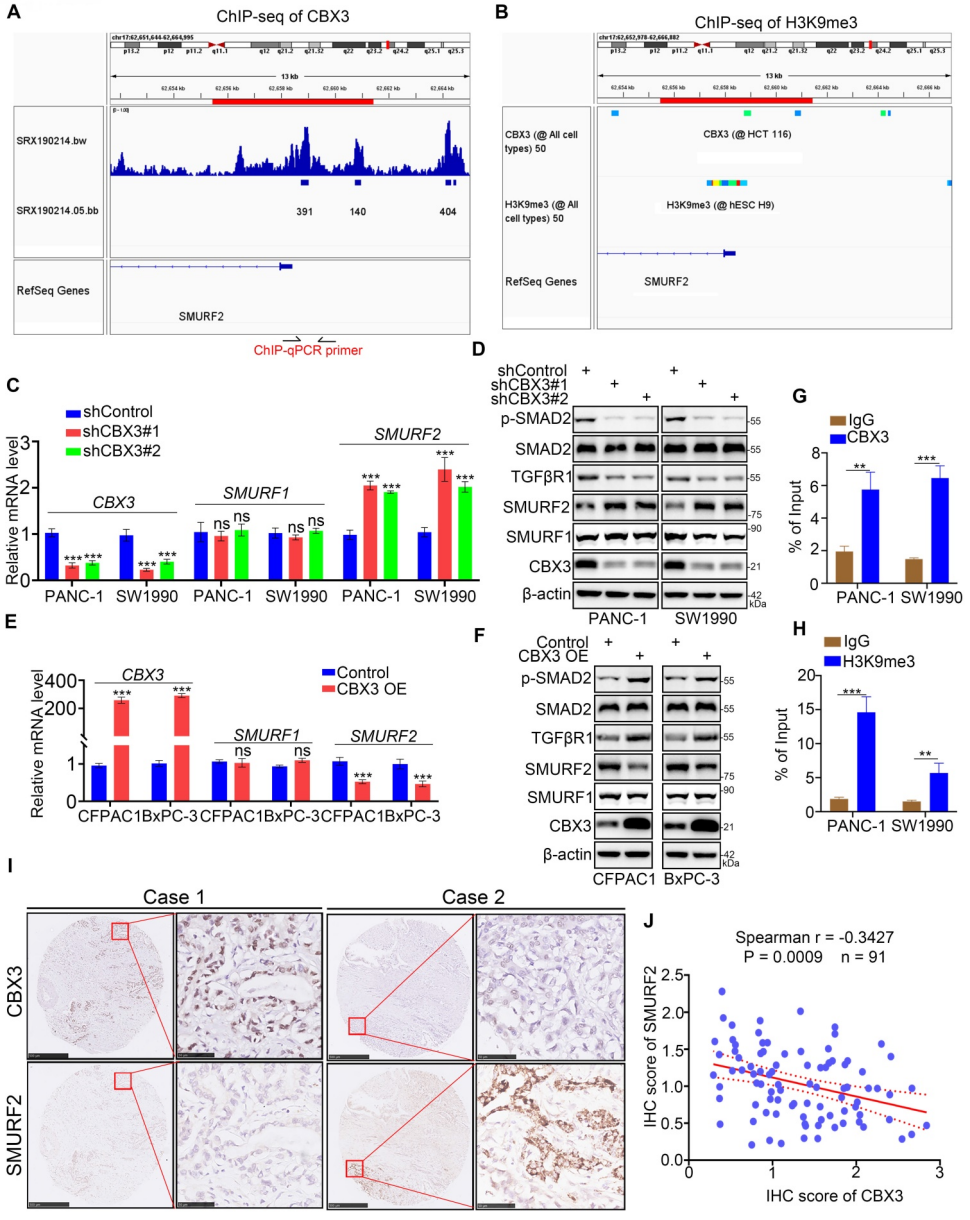
** CBX3 represses the SMURF2 expression in pancreatic cancer cells. (A and B)** ChIP-seq data of CBX3 and H3K9me3 on promoter region of SMURF2. **(C and D)** PANC-1 and SW1990 cells were transfected with CBX3 shRNAs for 72 hours. After puromycin selection, cells were harvested for RT-qPCR assay (C) and Western blot (D). **(E and F)** CFPAC1 and BxPC-3 cells were transfected with indicated constructs for 48 hours, and then were harvested for the RT-qPCR (E) and Western blot (F). **(G and H)** ChIP-qPCR of SMURF2 by using the CBX3 and H3K9me3 antibodies. **(I and J)** The tissue microarray of pancreatic cancer was stained with CBX3 and SMURF2. Typical IHC images stained with CBX3 and SMURF2 were shown in panel I. Correlation analysis of these two proteins was shown in panel J. Data in (C), (E), (G), (H) and (J) are presented as mean ± SD (n = 3). Not significant ^Ns^; P < 0.05 *; P < 0.01 **; P < 0.001 ***.

Then, we explored the mechanisms by which CBX3 activated the TGF-β signaling pathway. As CBX3 typically acts as a gene repressor, we assessed whether the expression of SMURF1 and SMURF2, two kinds of TGF-β signaling inhibitor, could be regulated by CBX3. ChIP-seq analyses revealed the presence of binding peaks of CBX3 and its corresponding function maker H3K9me3 22 in the promoter regions of SMURF1 and SMURF2 (Fig. 5A-B; Fig. S4A-B). CBX3 ablation simply upregulated the expression of SMURF2 but not SMURF1 in pancreatic cancer cells (Fig. 5C-D). Furthermore, SMURF2 but not SMURF1 was downregulated in CBX3-overexpressed pancreatic cancer cells (Fig. 5E-F). Moreover, SMURF2 has been reported to play a significant role in ubiquitin‐mediated proteasomal degradation of TGF‐β signaling components such as SMAD1, SMAD2, SMAD4, and TGFβR1 23. Consistently, we observed that CBX3 negatively regulated SMURF2 expression and SMURF2‐dependent TGFβR1 ubiquitination, thereby increasing the activation of TGF-β signaling (Fig. 5C-F; Fig. S4C). The ChIP-qPCR analysis also revealed the binding of CBX3 and H3K9me3 to the promoter region of SMURF2 in pancreatic cancer cells (Fig. 5G-H). Moreover, we detected the expression of CBX3 and SMURF2 in tissue microarrays from pancreatic cancer biopsies (n = 91). Immunohistochemical staining of tissues for CBX3 and SMURF2 demonstrated a negative correlation between CBX3 and SMURF2 (Fig. 5I-J). Taken together, our results indicate that CBX3 suppresses SMURF2 expression and activates the TGF-β pathway in pancreatic cancer.

### The YBX1/CBX3 axis promotes tumor growth via suppressing SMURF2 in pancreatic cancer

**Fig 6:**
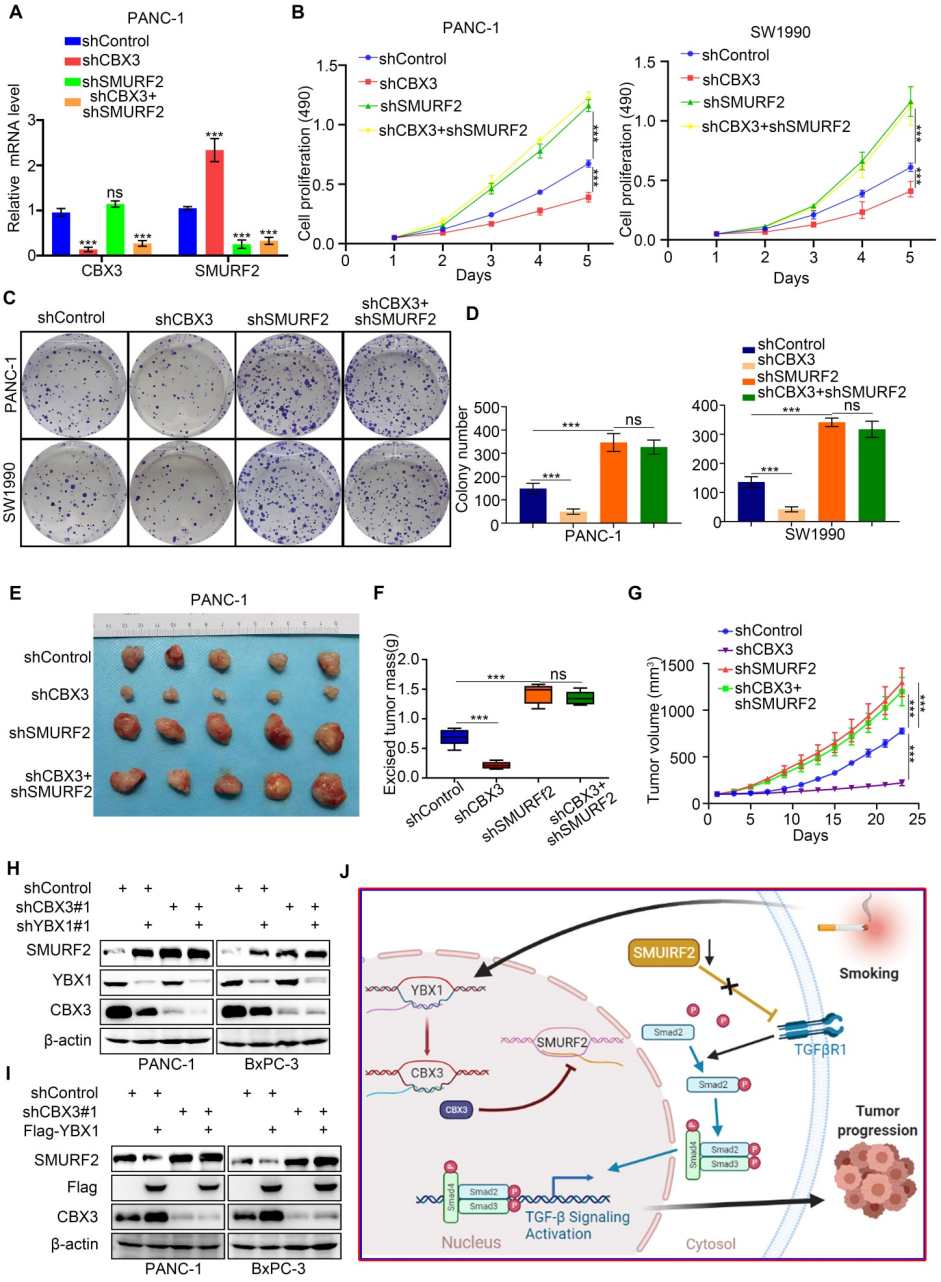
** The YBX1/CBX3 axis promotes tumor growth via suppressing SMURF2 in pancreatic cancer. (A-D)** PANC-1 and SW1990 cells were transfected with indicated shRNAs for 72 hours. After puromycin selection, cells were harvested for the RT-qPCR (A), MTS (B), and colony formation (C and D). **(E-G)** PANC-1 cells were transfected with indicated shRNAs for 72 hours. After puromycin selection, cells were subcutaneously injected into the nude mice. The image of tumor was shown in panel E. The tumor mass and tumor growth curve were demonstrated in panel F and G, respectively. Data are presented as mean ± SD (n = 5). **(H)** PANC-1 and BxPC-3 cells were transfected with indicated shRNAs for 72 hours. After puromycin selection, cells were harvested for the Western blot analysis. **(I)** PANC-1 and BxPC-3 cells were transfected with indicated shRNAs for 48 hours, and then were transfected with indicated constructs for another 24 hours. Cells were harvested for Western blot. **(J)** A hypothetic model depicted that CSE exposure-induced YBX1 overexpression contributed to the upregulation of CBX3, which inhibited SMURF2 expression to promote the progression of pancreatic cancer. Data in (A), (B) and (D) are presented as mean ± SD (n = 3). Not significant ^Ns^; P < 0.001 ***.

SMURF2 has been shown to suppress the ability of tumor formation of pancreatic cancer cells 24. As CBX3 was found to repress SMURF2 expression, we then evaluated the relevance of SMURF2 in CBX3-induced pancreatic cancer progression. To this end, we constructed pancreatic cancer cells with stable knockdown for CBX3, SMURF2, or both together (Fig. 6A; Fig. S4D). MTS and colony formation assays demonstrated that SMURF2 knockdown enhanced proliferation of pancreatic cancer cells; however, co-knockdown of SMURF2 and CBX3 could not reverse this process (Fig. 6B-D). Consistently, simultaneous silencing of CBX3 and SMURF2 in pancreatic cancer cells did not restore the ability of tumor growth in vivo (Fig. 6E-G). These data suggest that SMURF2 may be the key downstream effector of CBX3 in pancreatic cancer. As YBX1 regulated CBX3 expression in pancreatic cancer cells (Fig. 2), we knocked down and overexpressed YBX1 and found that YBX1 decreased the protein level of SMURF2 , which was attenutaed after co-knockdown of CBX3 (Fig. 6H-I). Collectively, these results demonstrate that YBX1 upregulates CBX3, which consequently represses SMURF2 and activates the TGF-β signaling pathway and promotes malignant progression in pancreatic cancer (Fig. 6J).

## Discussion

CBX family members are crucial parts of the PRC1 complex and epigenetic regulators modulating gene expression through chromatin modification 15. So far, eight members of the CBX family have been identified with a common chromodomain in the N-terminus 16. These eight CBX proteins were classified into two groups: (1) heterochromatin protein 1 (HP1) subgroup, including HP1β (CBX1), HP1γ (CBX3), and HP1α (CBX5); and (2) Polycomb CBX subgroup, the member of which contains a C-terminal polycomb repressor box to form the PRC1 complex 25. CBXs can act as tumor suppressors or oncoproteins. For instance, elevated expression of CBX1-3 was involved in poor progression in patients with ovarian cancer 25. Higher expression of CBX7 was correlated with better prognosis in hepatocellular carcinoma, whereas CBX1, CBX2, CBX3, CBX6 and CBX8 were identified as independent prognostic factors for poor overall survival 26. Similarly, high expression levels of CBX3-6 were correlated with poor prognosis in gastric cancer patients 27. It is worth noting that the mRNA levels of CBX1, CBX3, CBX5 and CBX8 were elevated in pancreatic cancer tissues compared with non-malignant pancreatic tissues. Importantly, only CBX3 was consistently overexpressed and correlated with poor prognosis in patients with pancreatic cancer. CBX3 was found to play a key role in cancer progression by suppressing the expression of SMURF2 in pancreatic cancer. SMURF1 and SMURF2 are important for the regulation of TGF-β pathway through mediating the ubiquitination and subsequent degradation of SMADs 28. As a pleiotropic cytokine, TGF-β may have an influence in disease progression of pancreatic cancer 29-31. SMURF1 and SMURF2 have overlapping but also distinct substrates and regulatory mechanisms, despite the high homology in their WW domains 32. SMURF1 was found to promote tumor invasion in pancreatic cancer 33. In contrast, SMURF2 suppressed the development and progression of pancreatic cancer 24, 34. In this study, we demonstrated that CBX3 downregulated SMURF2 but not SMURF1, consistent with the biological function of CBX3 in pancreatic cancer. Future studies are required to explore novel mechanisms by which CBX3 promotes pancreatic cancer progression.

Cigarette smoking is a well-known environmental risk factor for pancreatic cancer. The analysis conducted by the International Pancreatic Cancer Case-Control Consortium (PanC4) demonstrated that current cigarette smoking was involved in a two-fold increased risk of pancreatic cancer 35. Another analysis of 10 population-based cohort studies in Japan also indicated that cigarette smoking was associated with 1.59- and 1.81-fold increased risk of pancreatic cancer in men and women, respectively 36. Accumulating evidence shows that various pathways are responsible for the development of pancreatic cancer in response to smoking 37. Smoking has been shown to induce mutations in KRAS, SMAD4, and TP53, which are major oncogenic drivers in pancreatic cancer 21, 38, 39. Recently, Zhang et al. reported that CSE exposure enhanced m6A modification catalyzed by overexpressed methyltransferase-like 3 (METTL3), which promoted pancreatic cancer progression 40. However, the mechanisms underlying the development and progression of smoking-related pancreatic cancer remain unclear.

Our findings revealed that cigarette smoking induced the overexpression of CBX3 in pancreatic cancer cells and tissues, which consequently suppressed the expression of SMURF2 and enhanced the malignant potential of pancreatic cancer. As a tumor promoter upregulated by smoking, CBX3 may be an ideal target for clinic treatment of pancreatic cancer. Nevertheless, no selective small molecule inhibitors of CBX3 are currently available. Therefore, understanding the mechanisms regulating CBX3 expression and function may facilitate the development of small molecule inhibitors of CBX3. Long non-coding RNAs, including LINC00998 40, SNHG17 41 and KCNQ1OT1 42, as well as microRNAs (miRs), such as miR-30b 43, miR-375 41, miR-1265 44 and miR-29a-3p 42, have been found to regulate the expression of CBX3. Here, our study showed that CBX3 overexpression after CSE treatment was mediated by YBX1. Whereas, there were no specific small molecule inhibitors targeted to the above molecules involved in regulating the expression CBX3. Thus, the further knowledge of regulation of CBX3 in transcriptional and post-transcriptional modifications are required for identifying ideal targeted inhibitors and improving clinic outcomes.

## Conclusion

In conclusion, cigarette smoking upregulated CBX3 in pancreatic cancer cells and tissues and this abnormal upregulation was associated with poor prognosis. Moreover, CSE-induced YBX1 overexpression contributed to the upregulation of CBX3 in pancreatic cancer cells. CBX3 promoted malignant progression partly by inhibiting the expression of SMURF2 and activating the TGF-β pathway in pancreatic cancer. Therefore, the YBX1/CBX3/SMURF2 signaling axis may be considered as a promising target for the treatment of smoking-related pancreatic cancer.

## Supplementary Material

Supplementary methods, figures and tables.

## Abbreviations

CBX3: Chromobox protein homolog 3
SMURF2: SMAD specific E3 ubiquitin protein ligase 2
YBX1: Y-box-binding protein 1
PRC1: Polycomb repressive complex 1
CPTAC: Clinical Proteomic Tumor Analysis Consortium
ICGC: International Cancer Genome Consortium
TCGA: The Cancer Genome Atlas
CSE: Cigarette smoke extract
ChIP: Chromatin immunoprecipitation
TF: Transcriptional factors
shRNAs: short-hairpin RNAs
GSEA: Gene set enrichment analysis
AU: Australia
CN: Canada
METTL3: Methyltransferase-like 3
TIMER2.0: Tumor immune estimation resource 2.0
miR: microRNA
