# Supplementary material for: CBX3 Regulated By YBX1 Promotes Smoking-induced Pancreatic Cancer Progression via Inhibiting SMURF2 Expression
Source: Int J Biol Sci. 2022 May 13;18(8):3484–97. doi: 10.7150/ijbs.68995 (PMC9134897; doi:10.7150/ijbs.68995)
Supplement: Supplementary file 1 — Supplementary methods, figures and tables. [file ijbsv18p3484s1.pdf]

Supplementary figure 1

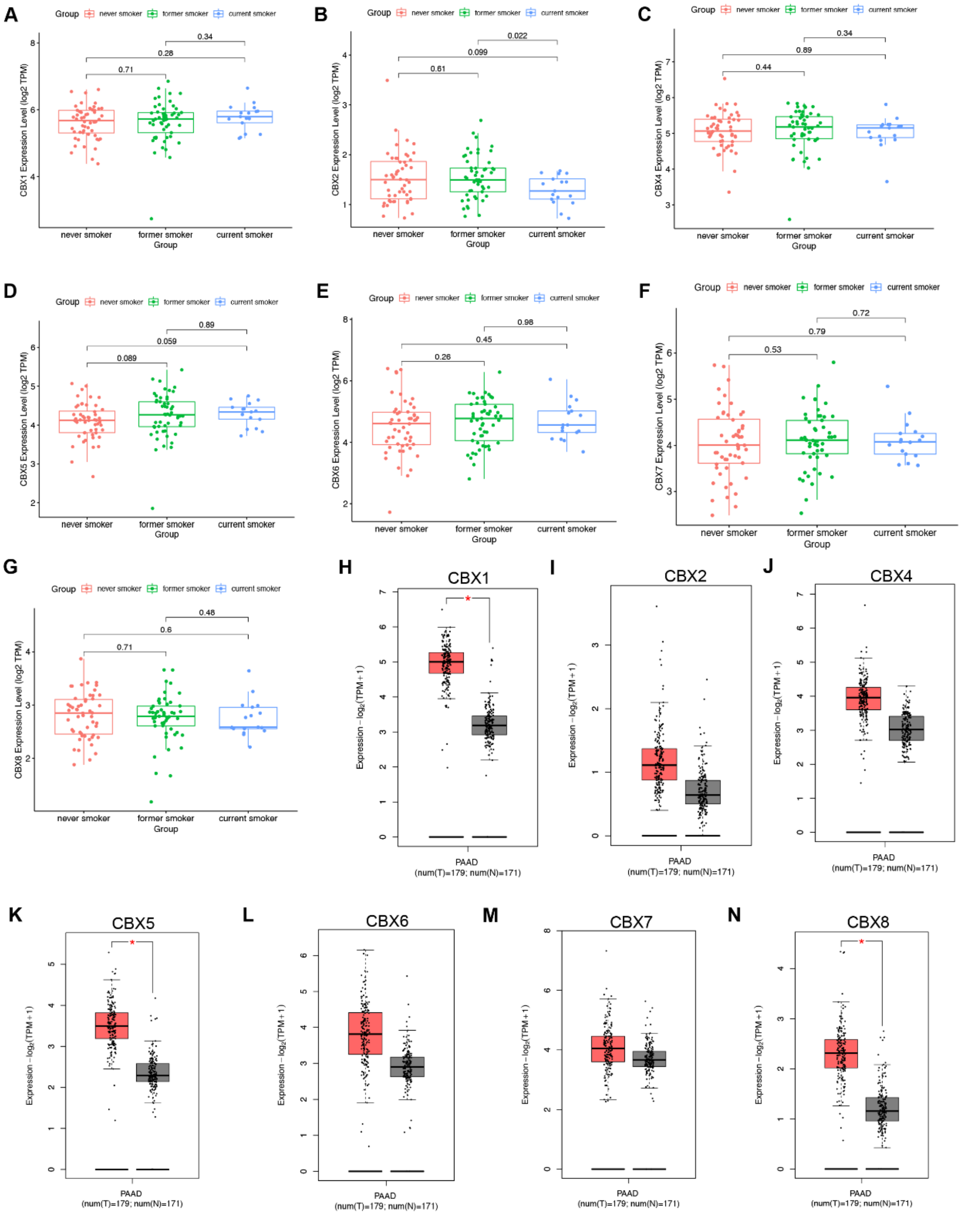

Supplementary Fig. 1. (A-G) Box plot showed the expression level of CBX1-2 and CBX4-8 in TCGA-PAAD dataset with distinct smoking status (never smoker, former smoker, and current smoker). **(H-N)**, the expression level of CBX1-2 and CBX4-8 in the non-tumor pancreatic tissues and pancreatic cancer tissues by using the TCGA dataset.

Supplementary figure 2

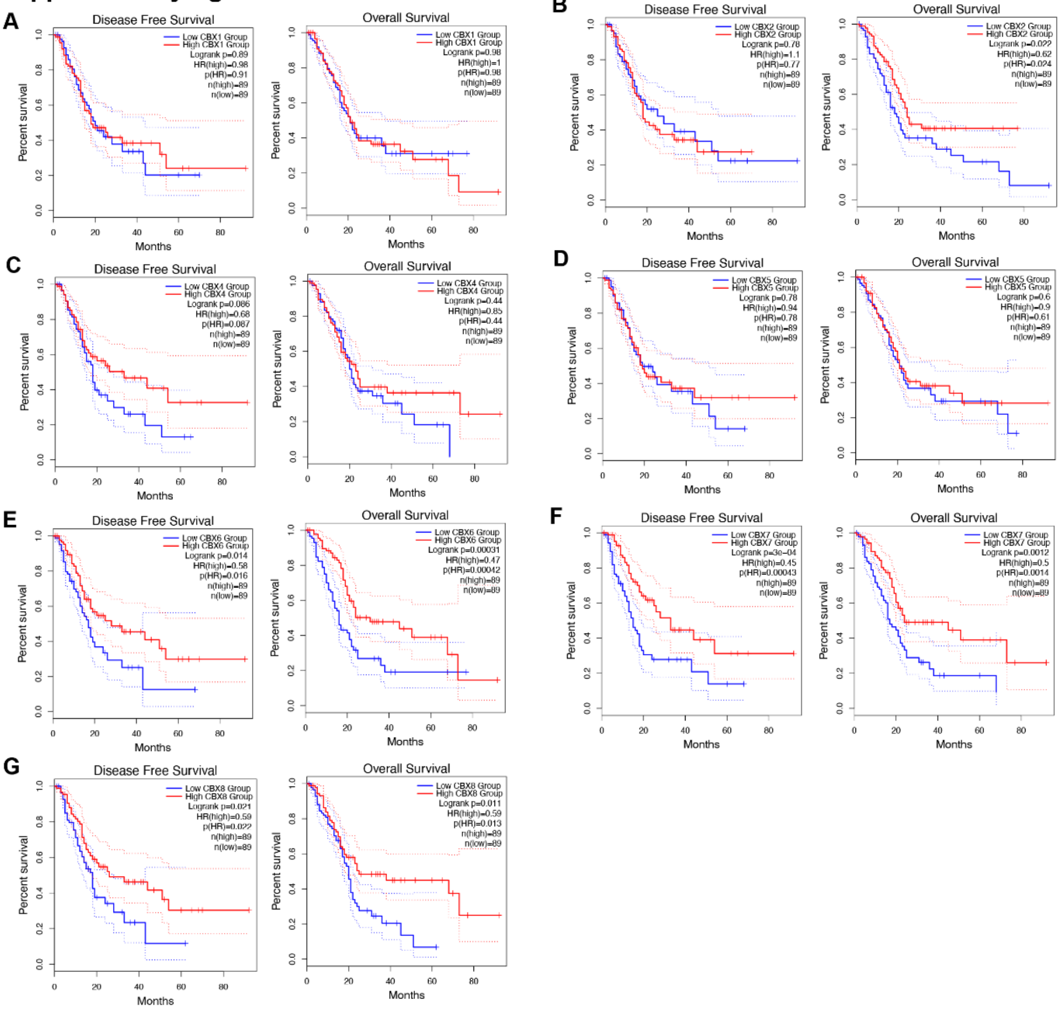

Supplementary Fig. 2.

The disease-free and overall survival of CBX1-2 and CBX4-8 in the patients with pancreatic cancer by using the GEPIA web tool.

Supplementary figure 3

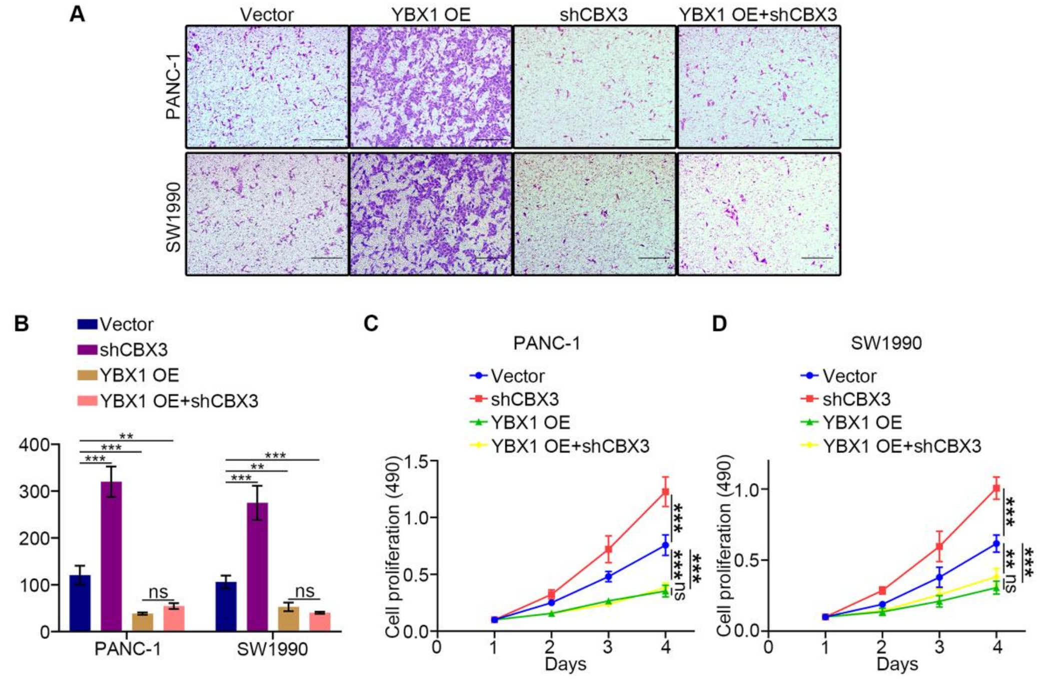

Supplementary Fig. 3

(A-D) PANC-1 and SW1990 cells were transfected with indicated shRNAs and/or overexpression plasmid (vector, YBX OE; shCBX3; YBX OE+shCBX3), and then were harvested for Transwell assay (A and B) and MTS assay (C and D). One-way ANOVA followed by Turkey’s multiple comparisons post hoc test was applied for the statistical analysis. not significant ^{ns}; P < 0.01 **; P < 0.001 ***.

Supplementary figure 4

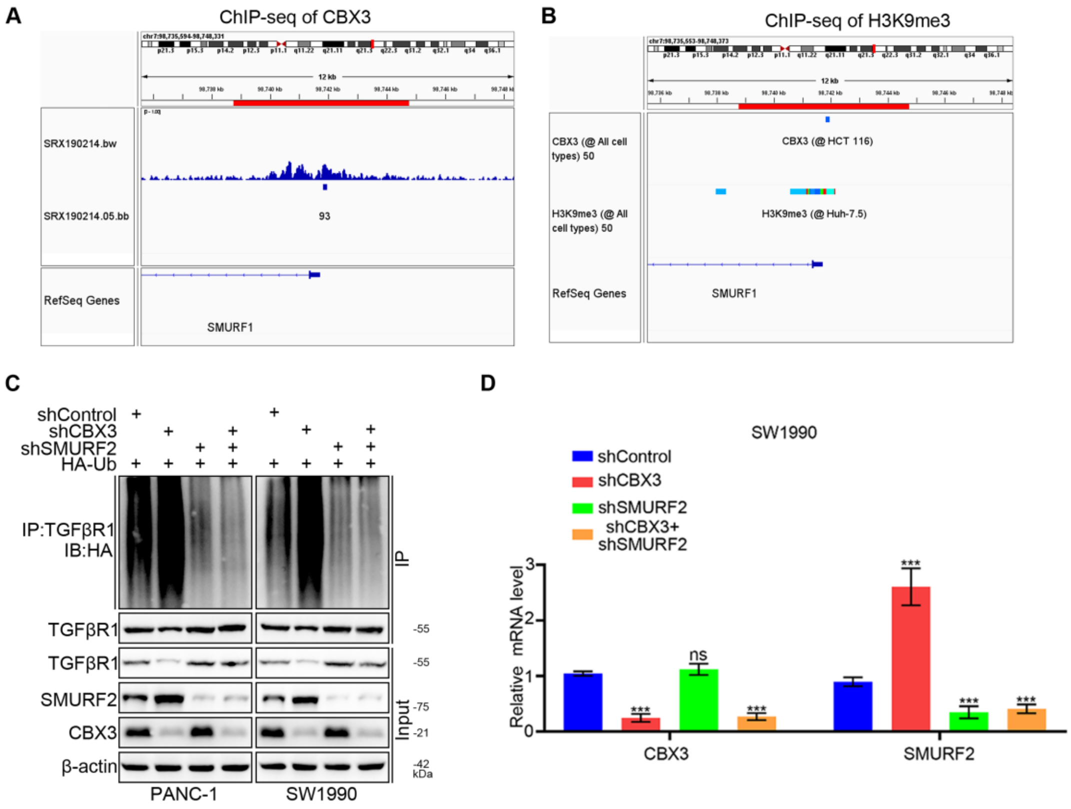

Supplementary Fig. 4

(A and B) the ChIP-seq data of CBX3 and H3K9me3 on the promoter region of SMUFR1**. (C)** Co-IP to detect TGFβR1 ubiquitination. PANC-1 and SW1990 cells were transfected with the indicated shRNAs and HA-Ub for 72 hours. The protein lysates were immunoprecipitated with an anti- TGFβR1 antibody and immunoblotted with the indicated antibodies. **(D)** SW1990 cells were infected with indicated shRNAs for 72 h. After puromycin selection, cells were harvested for the RT-qPCR assay (D). Data are presented as mean ± SD (n = 3). One-way ANOVA followed by Turkey's multiple comparisons post hoc test was applied for the statistical analysis. not significant ^{ns}; P < 0.01 **; P < 0.001 ***.

Table S1. The primer sequences for RT-qPCR.

Gene (Human)	Forward primer (5′ - 3′)	Reverse primer (5′ - 3′)
GAPDH	ATGACAATGAATACGGCTACAGCA	GCAGCGAACTTTATTGATGGTATT
CBX3	GAGATGCTGCTGACAAACCA	TATTTGCCTCTTTCGCCAGC
YBX1	AAGTGATGGAGGGTGCTGAC	TGACCTTGGGTCTCATCTCC
SMURF1	TCCTCAGACACGAACTGTCG	ATTCAGCATTTCATGGCACA
SMURF2	TAGCCCTGGCAGACCTCTTA	AATACACCTGGCCTTGTTGC

Table S2. The shRNA sequences.

shCBX3-1	5′-CCGGCTGGCGAAAGAGGCAAATATGCTCGAGCATATTTGCCTCTTTCGCCAGTTTTTG-3′
shCBX3-2	5′- CCGGCGACGTGTAGTGAATGGGAAACTCGAGTTTCCCATTCACTACACGTCGTTTTT-3′
shYBX1 #1	5'-CCGGCACCGCTTTAGTTTGTAAGTATTTATTCAAGAGATAAATACTTACAAACTAAAGCTTTTTG-3'
shYBX1 #2	5'- CCGGCACCGCTATTTATAGGACCCTTAGCTTCAAGAGAGCTAAGGGTCCTATAAATAGCTTTTTT-3'
shSMURF2 #1	5'-CCGGCACCGCAGTCGTTTATTTGATAACGTTCAAGAGACGTTATCAAATAAACGACTGCTTTTTG-3'
shSMURF2 #2	5'-CCGGCACCGCAGACCTCTTAGCTGCTTTGTTCAAGAGACAAAGCAGCTAAGAGGTCTGCTTTTT-3'

Table S3. The primer sequences for ChIP-qPCR.

Gene	Forward primer (5′ - 3′)	Reverse primer (5′ - 3′)
CBX3 primer I (YBX1)	TGGCGCTGTAGTGAGAACTG	GTACGCATTTGGCGTAAGGT
CBX3 primer O (YBX1)	GAGCCTCTGAAGCCATGTTT	AGGGACGAAGGTGGTTTTCT
SMURF2 (CBX3)	CAGGGCCAACTGCATTTTAT	TCTCCTCCACCAGGTGAGTC

Supplementary Material and Methods

Public datasets for data mining and bioinformatics analysis

1） TCGA-LUAD：Transcriptome data and clinical information of LUAD patients were obtained from the GDC data portal (https://portal.gdc.cancer.gov/). Data from 522 LUAD patients and 60488 genes were acquired. Of these patients, 59 samples had matched normal tissues.

2） TCGA-LUSC： Transcriptome data and clinical information of LUSC patients were obtained from the GDC data portal (https://portal.gdc.cancer.gov/). Data from 504 LUSC patients and 60488 genes were acquired. Of these patients, 49 samples had matched normal tissues.

3） TCGA-PAAD：Transcriptome data and clinical information of PAAD patients were obtained from the GDC data portal (https://portal.gdc.cancer.gov/). Data from 150 PAAD patients and 60488 genes were acquired. Of these patients, 4 samples had matched normal tissues.

4） ICGC-PACA-AU: Transcriptome data and clinical information of PAAD patients were obtained from the ICGC database (https://dcc.icgc.org/). Data from 91 donors were acquired.

5） ICGC-PACA-CA: Transcriptome data and clinical information of PAAD patients were obtained from the ICGC database (https://dcc.icgc.org/). Data from 234 donors were acquired.

6） CPTAC-PDAC: Proteomics data and clinical information of PAAD patients were obtained from the CPTAC data portal (https://proteomics.cancer.gov/programs/cptac). Data from 135 PAAD patients and 60483 genes were acquired. Of these patients, 8 samples had matched normal tissues.

Identification of smoking-related CBX family genes

Since nonsmokers are underrepresented in smoking-related cancer patients, smoking-related genes were mainly identified between current smokers and former smokers in this study. We also required that the smoking-related genes were differentially expressed in TCGA-LUAD, TCGA-LUSC and TCGA-PAAD datasets consistently.

Survival analysis

PAAD patients were divided into two groups according to the median expression level of the key gene. The differences in RFS and OS between the high and low expression groups were evaluated by the Kaplan–Meier method, followed by a log-rank test.

GSEA for the key gene

PAAD patients were first divided into two groups according to the median expression level of the key gene. Then, differential expression analysis was applied between the high and low expression groups. Input genes for GSEA were sorted by their logFC values. Signaling pathways activated or suppressed by the key gene were decided by the NES value derived from GSEA.

Upstream/Downstream targets of the key gene

Upstream/Downstream targets of the key gene were determined by ChIP-seq and correlation analysis. Binding site in the targeted gene promoter of the key protein or methylation were obtained from ChIP-Atlas database (https://chip-atlas.org/). Correlation analysis between gene/protein and targeted genes was applied to further validate the ChIP-seq results ($|r| \geq 0.30$ & $P < 0.05$).

Statistical analysis and visualization

Microsoft R Open v4.0.2 was used for data mining, bioinformatics analysis and visualization in transcriptomics data. IGV v2.9.0 was used for analysis and visualization of ChIP-seq data.
